# Development of Lupus Erythematosus Tumidus During the Course of Systemic Sclerosis

**DOI:** 10.7759/cureus.18064

**Published:** 2021-09-17

**Authors:** Constantine N Logothetis, Nikifor K Konstantinov, Michael D Reyes, N. Suzanne Emil, Antonios H Tzamaloukas

**Affiliations:** 1 Internal Medicine, University of South Florida, Tampa, USA; 2 Dermatology, University of Minnesota School of Medicine, Minneapolis, USA; 3 Pathology, Raymond G. Murphy Veterans Affairs (VA) Hospital, Albuquerque, USA; 4 Internal Medicine, University of New Mexico School of Medicine, Albuquerque, USA

**Keywords:** lupus erythematosus tumidus, systemic sclerosis, cutaneous lupus erythematosus, interferon-mediated disease, anti-nuclear antibodies

## Abstract

A man with systemic sclerosis (SS), manifested by characteristic skin lesions, gastro-esophageal reflux disease, and pulmonary fibrosis producing progressive respiratory failure, and a positive antinuclear antibody consistent with reactivity to fibrillarin, developed skin lesions with the clinical and histological characteristics of lupus erythematosus tumidus (LET) 10 years after the diagnosis of SS. His respiratory failure progressed and he expired from sepsis after tracheal intubation and mechanical ventilation two years after developing LET. The association of SS and LET, not described until now, raises questions about its pathogenesis and its prognostic significance.

## Introduction

Various types of autoimmune diseases can overlap in the same patient. Distinct serological markers may characterize the clustering of autoimmune diseases [1]. Clustering is seen often for certain types of autoimmune diseases, but not for others. Clustering of SLE or cutaneous lupus erythematosus (CLE) with systemic sclerosis (SS) is rare. A survey of 118 Italian patients with SS found that 38 patients (32.2%) had also one or more other autoimmune diseases [2]. In this survey, the autoimmune diseases most frequently overlapping with SS were autoimmune thyroiditis (17 patients) and primary Sjögren’s syndrome (12 patients), while clustering of SS and discoid lupus erythematous was found in only one patient [2]. Lupus erythematosus tumidus (LET) is an uncommon variety of CLE described early in the 20th century [3]. LET is manifested clinically by non-scarring erythematous plaques on skin areas exposed to the sun and histologically by perivascular and periadnexal lymphocytic infiltrates and deposits of mucin in the interstitium, classically without epidermal involvement [3]. To our knowledge, clustering of SS and LET has not been reported until now. We present a patient who developed LET 10 years after he was diagnosed with limited diffuse SS. This case generated important questions about the factors affecting the coexistence of autoimmune diseases and the prognostic significance of such clustering.

This article was previously presented as a meeting abstract at the 2019 American Federation for Medical Research and Participating Societies Western Medical Research Conference in Carmel, California, in January 2019.

## Case presentation

**Figure 1:**
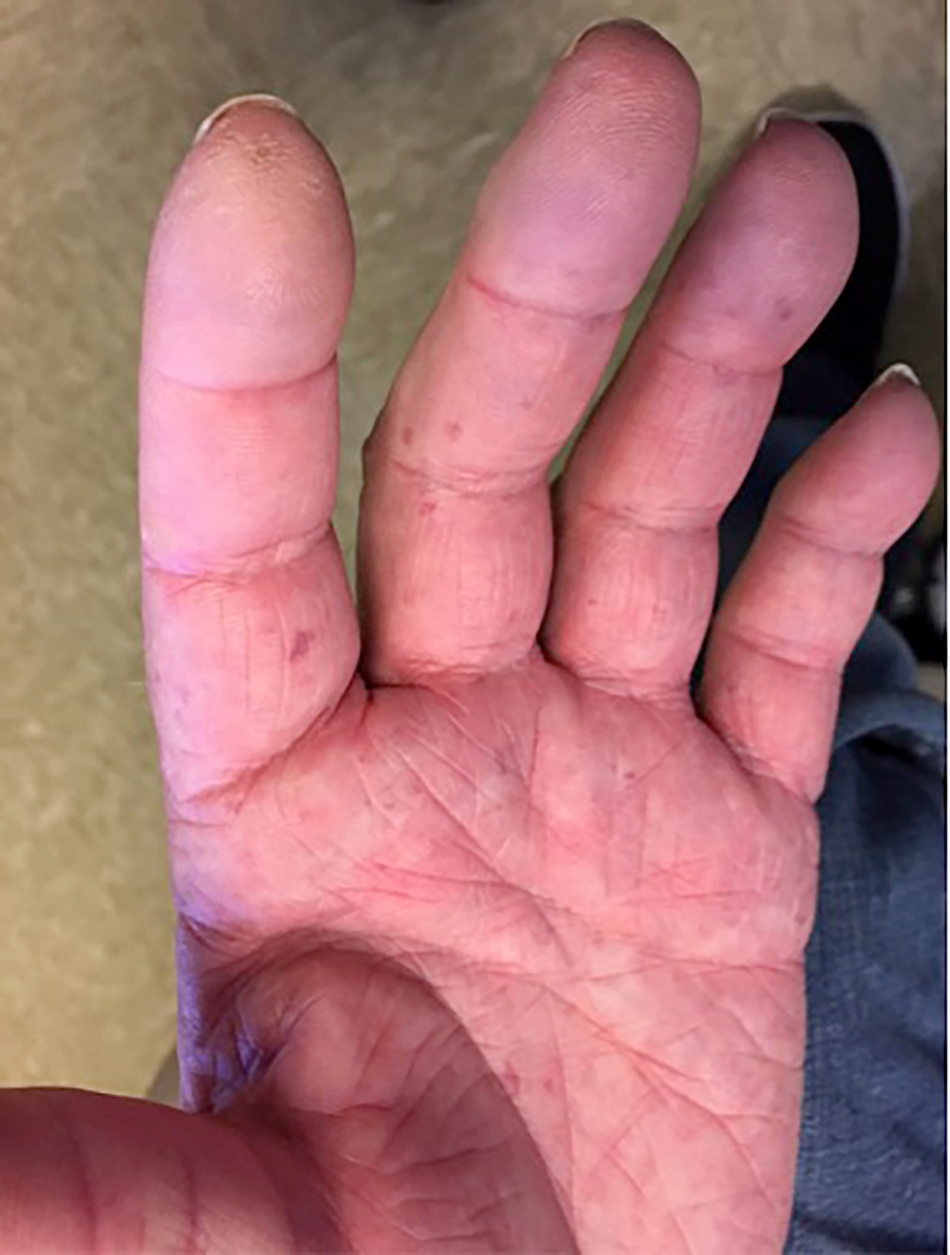
Patient’s left-hand palmar surface with prominent telangiectasia and digital pitting.

A 58-year-old man with a history of coronary artery disease and coronary bypass surgery in 2005 developed sclerodactyly and digital pitting scars in multiple digits (Figure 1), palmar and facial telangiactasias, plus tightness of lips and skin around the neck. Capillaroscopy revealed capillary nailfold dropout. He also had gastroesophageal reflux disease (GERD). At that time, he was diagnosed with limited diffuse SS. Over the ensuing years, the sclerodactyly progressed; in 2011, he developed exertional dyspnea and was diagnosed with pulmonary fibrosis. Testing for antinuclear antibodies (ANA) by indirect immunofluorescence (IIF) technique using Hep2 cells produced clumpy nucleolar and coilin body staining pattern in interphase cells at a dilution of 1:640, and approximately 34-kDa band on Western blotting, consistent with reactivity to fibrillarin (data not shown). Rheumatoid factor, antibodies for double-stranded DNA, RNP, Smith, Scl-70, centromere, SS-A, SS-B, Jo-1, and cyclic citrullinated peptide were negative and serum complement levels were in the normal range.

**Figure 2:**
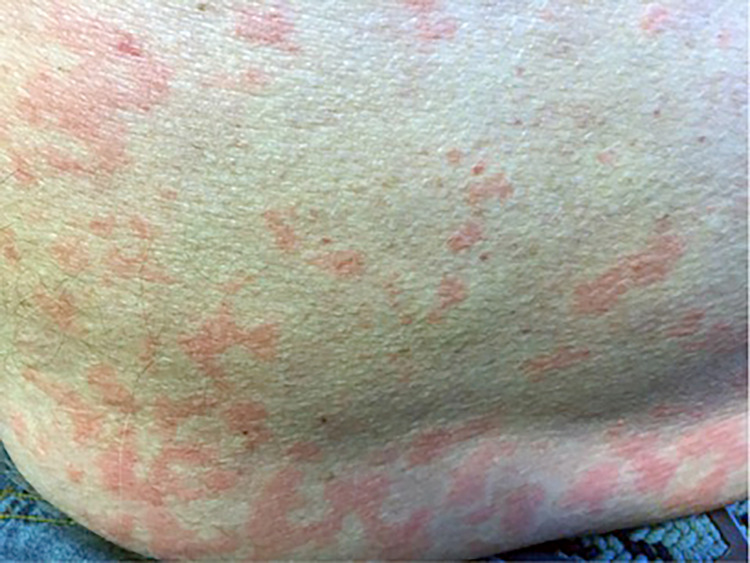
Left lower back eruption consisting of non-scaly pink thin plaques in a livedo reticularis-like pattern.

His SS and pulmonary fibrosis had been stable without the use of immune modulators until 2014 when he developed pulmonary hypertension and was placed on hydroxychloroquine. In 2015, he developed photosensitive erythematous lesions located on the upper part of his trunk and neck. The eruption consisted of succulent, non-scaly pink elevated plaques on the trunk, which seemed to resemble a livedo reticularis-like pattern (Figure 2).

**Figure 3:**
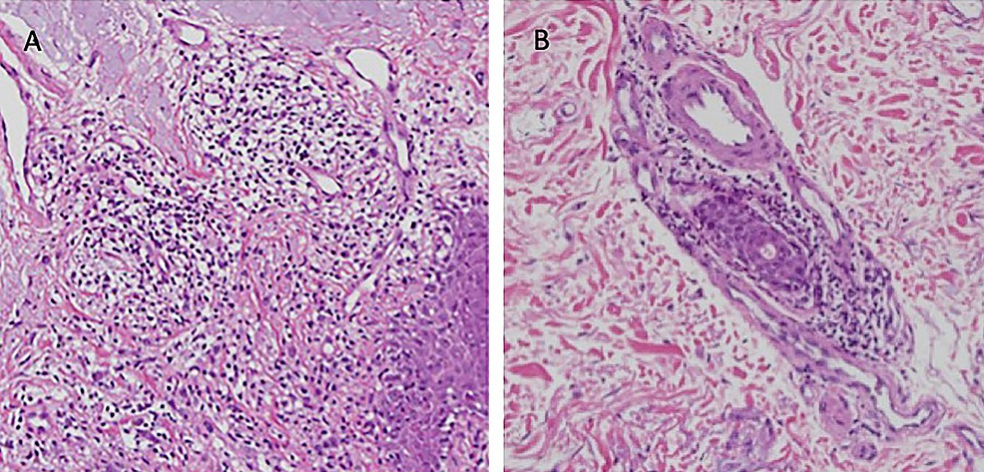
Punch biopsy of the left lower back. There is a perivascular lymphocytic infiltrate with interstitial mucin deposition in the dermis (A) (hematoxylin and eosin [H&E], original magnification x40) and periadnexal lymphocytic infiltrate (B) (H&E, original magnification x40).

Biopsy of the lesion of the left lower back showed a superficial and deep periadnexal and perivascular lymphocytic infiltrate without follicular plugging or interface changes in the dermal-epidermal junction (Figures 3A, 3B).

Mucin deposits were present. Direct immunofluorescence showed focal granular deposits of IgM, C3, and C5b-9 more pronounced at the adnexal than epidermal basement membrane and the lower third of the skin strata. The diagnosis of LET was based on the clinical and histological characteristics of the lesion.

The patient’s pulmonary hypertension was subsequently treated with endothelin receptor antagonists. Macitentan (Opsumit), which was used first, was replaced by epoprostenol (Veletri) because the use of macitentan was linked to the development of a stuffy nose and fluid retention. However, there was no change in the rash after changing the pharmacological therapy. Diffuse erythematous thin papules coalescing into widespread bright salmon-red plaques were present over the chest, back, face, arms, legs, and buttocks. A sclerotic, purple-hued infiltrative plaque was noted over the posterior neck. The features of LET improved with topical pimecrolimus cream (Elidel). However, pulmonary hypertension progressed with worsening dyspnea and ambrisentan (Letairis) was started in February 2016. In April 2017, the patient’s respiratory failure required tracheal intubation and mechanical ventilation. He subsequently expired from septic shock.

## Discussion

Cutaneous manifestations are prominent in both SS and lupus erythematosus (LE). Skin thickening of the fingers extending proximally to the metacarpophalangeal joints, without any other manifestations, classifies a patient as having SS [4]. Similarly, skin manifestations, including malar rash, photosensitivity, discoid lesions, and oral ulcers, are the only four criteria leading to the diagnosis of LE in patients without any other organ involvement and some patients with SLE [5]. Photosensitivity and smoking [6] are established environmental risk factors for the development of various types of CLE.

Skin is the most common organ involved in LE. The importance of CLE is underlined by the finding that skin manifestations are present in 70% to 85% of patients with LE and are the first manifestation of LE in approximately 25% of the patients [7]. An index comprising a set of criteria identifying the extension and severity of CLE manifestations was developed and revised [8]. Cutaneous lesions of LE are classified into lupus-specific and not lupus-specific [9]. Non-lupus-specific skin lesions are associated with various manifestations of SLE more frequently than lupus-specific lesions [10]. Lupus-specific cutaneous lesions are classified as acute, subacute, and chronic [10]. LET is routinely classified as a chronic variety of cutaneous LE [9]. However, some investigators classify LET as intermittent CLE.

The clinical features of LET consist of the development of nonscarring, erythematous, succulent plaques with no epidermal changes in the exposed areas of the skin [3,11,12]. The histological characteristics of LET consist of deposition of lymphocytes in perivascular and periadnexal areas, mucin deposition in the interstitium, absence or minimal lesions in the dermo-epidermal junction, and preservation of the epidermis [3,11,12]. In one study, immunohistochemical staining disclosed that approximately 80% of the lymphocytes in the infiltrates were T cells, with a three times higher number of helper cells over cytotoxic cells [13]. The distinction between cutaneous lymphoma and lymphocytic infiltrate of the skin in LET and other forms of CLE requires careful clinical and histopathological evaluation. Mucin deposition in the interstitium, which was reported in all studies of SLE, may vary between slight and intense. Direct immunofluorescence shows no deposits [3] or deposits similar to those observed in the patient of this report [12].

Spontaneous clearing of LET is seen in a few patients [11]. Antimalarial agents, including chloroquine and hydroxychloroquine, or topical steroids improved significantly or cleared LET in several studies [3,12]. Topical tacrolimus in conjunction with hydroxychloroquine [14] and rituximab [15] have been used in patients with LET resistance to antimalarials.

In the reported series of patients, LET was an independent clinical entity not associated with SLE or other forms of CLE [8]. Compared to discoid LE, LET was found to exhibit lower activity and severity scores, more abundant mucin deposition, and less dermo-epidermal junction damage. However, LET has been observed in individuals with SLE and other forms of DLE.

The co-existence of autoimmune diseases is not unusual, and the likelihood that a patient with an autoimmune disorder will acquire another one is high. Yet despite intense genetic and population studies on autoimmunity, the patterns of autoimmune clustering across the spectrum of these diseases remains elusive: inheritance of an “at risk” genotype, environment-susceptible phenotype, and random-chance have all been suggested, but not proven [16,17]. Recently, a study of the most prominently overexpressed genes in SS and SLE were found to belong in the same spectrum of interferon (IFN)-mediated diseases [18] and a subset of SS patients had “lupus-like” high IFN-inducible gene expression that correlated with the presence of certain autoantibodies [18]. The patient we describe had a strong autoimmune response to only U3 ribonucleoprotein (RNP)/fibrillarin. The immunodominant peptide sequence of fibrillarin, GRDLINLAKKRTNII, is homologous to viral protein sequences from the Mimiviridae and Phycodnaviridae families [19], which may suggest a link between HLA alleles, autoantibodies, and environmental triggers that drive the autoimmune response in susceptible individuals [19]. It has been suggested that clusters of autoimmune diseases in the same patient may depend on the target cell/affected organ, gender, ancestry, trigger factors, and age of onset [20].

## Conclusions

The association of SS and LET has not been reported before. Cutaneous lupus has been tied to risk for coexisting autoimmune conditions, but in our case, the SS manifestations preceded the development of LET by several years. Accumulation of similar cases may clarify if the onset of LET in a patient with full-blown SS has prognostic significance, and may provide opportunities for a better understanding of its pathogenesis.
